# Suspended Lithium Nitrate‐Based Electrolytes: Electrostatic Interactions for Mutually Rewarding Interface Optimization Strategies

**DOI:** 10.1002/advs.202416656

**Published:** 2025-03-20

**Authors:** Wenjing Zhang, Zhenguo Zhang, Hongtao Zhang, Yang Luo, Xinjian Liu, Zhonghao Rao

**Affiliations:** ^1^ Hebei Engineering Research Center of Advanced Energy Storage Technology and Equipment School of Energy and Environmental Engineering Hebei University of Technology Tianjin 300401 China; ^2^ Hebei Key Laboratory of Thermal Science and Energy Clean Utilization School of Energy and Environmental Engineering Hebei University of Technology Tianjin 300401 China; ^3^ School of Materials Science and Engineering Hebei University of Technology Tianjin 300401 China; ^4^ State Key Laboratory of Reliability and Intelligence of Electrical Equipment Hebei University of Technology Tianjin 300401 China

**Keywords:** interface optimization, lithium metal batteries, suspension electrolyte, wide temperature range

## Abstract

Designing a stable electrode‐electrolyte interface (EEI) is critical for developing lithium metal batteries with high energy density, enhanced safety, and broad applicability. Lithium nitrate (LiNO_3_) is an attractive sacrificial additive for lithium metal anode, while its poor solubility in high‐voltage‐resistant ester/nitrile electrolytes severely limits its utility. To solve it, a novel suspension electrolyte strategy is proposed that uniformly disperses LiNO_3_ particles in an ester/nitrile mixed electrolyte to stabilize the electrode interface. The suspended LiNO_3_ particles exhibit dual functionality: LiNO_3_ enhances the compatibility between the electrode and the electrolyte by affecting the Li^+^ solvation environment and preferentially adsorb on the electrode surface; moreover, the in situ formed LiN_x_O_y_‐rich EEI by LiNO_3_ decomposition with accelerated Li⁺ transport kinetics, effectively suppresses parasitic reactions and improves rate performance. The optimized electrolyte makes Li||NCM523 battery run stably for 100 cycles with a high capacity retention of 90.05% at 60 °C and stably operated at low temperature (‐10 °C). Moreover, the electrolyte shows excellent electrochemical stability at a high‐voltage of 4.5 V. This work presents a dual‐strategy advancement featuring wide‐temperature electrolyte formulation and precision interface engineering, synergistically achieving high‐specific‐energy lithium metal batteries.

## Graphical abstract

Suspended LiNO_3_ particles have a dual function: LiNO_3_ enhances electrode‐electrolyte compatibility by influencing the Li^+^ solvation environment. At the same time, LiNO_3_ adsorbed on the electrode surface can decompose in situ to form LiN_x_O_y_‐rich EEIs with the kinetic property of accelerated Li^+^ transport. A stable electrode‐electrolyte interphase structure is established, which improves the cycling recovery in a wide temperature range.

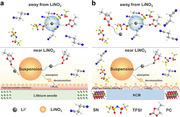

## 1 Introduction

Lithium─ion batteries (LIBs) power virtually renewable electrochemical energy storage devices such as portable electronics and electric vehicles (EVs).^[^ ^1^, ^2^, ^3^ ^]^ However, with the rapid development of EVs, current LIBs featuring graphite as an anode make it difficult to meet the demand for high energy density (the theoretical specific energy density of LIB is limited to 350 Wh kg^−1^).^[^ ^4^, ^5^, ^6^ ^]^ There is an urgent need to develop the next generation of high‐energy storage materials. Among numerous anode materials, lithium metal is a promising alternative to graphite anode due to its ultra‐high theoretical specific capacity (3860 mAh g^−1^) and extremely low redox potential (‐3.040 V vs SHE).^[^ ^7^, ^8^, ^9^ ^]^ Thus, matching it to high‐voltage cathode materials is expected to achieve the target of high specific energy.

However, lithium metal batteries (LMBs) still face the challenges of electrode‐electrolyte interface (EEI) side reactions as well as capacity degradation under extreme conditions such as high temperature and high voltage. On one hand, side reactions between lithium and the electrolyte are unavoidable due to the high reactivity of lithium, leading to electrolyte depletion and the formation of a solid electrolyte interface (SEI). In addition, high cut‐off voltage induces oxidative decomposition of electrolytes at the cathode surface, destabilizing the cathode‐electrolyte interphase (CEI) and leading to transition metal dissolution. Although commercially carbonate‐based electrolytes show good compatibility with existing LIBs, they are poorly suited to high‐voltage cathode materials and lithium metal anodes due to the continuous interfacial side reactions. Therefore, developing new electrolytes is advised to construct stable EEI, that are compatible with high energy density LMBs and thus achieve long cycle life under extreme conditions.

Recent research has found succinonitrile (SN) exhibits excellent ionic conductivity, high melting point, and good compatibility with high‐voltage cathode materials,^[^ ^10^ ^]^ which have potential applications for matching Li metal anode and high‐voltage cathode materials. However, serious side reactions between SN and lithium metal persist, which limits its wide application. Therefore, it is necessary to preferentially form a stable EEI on the electrode surface to inhibit interfacial reactions. Lithium nitrate (LiNO_3_), an effective additive commonly used in Li||S batteries,^[^ ^11^ ^]^ preferentially reacted with lithium to generate excellent lithium ion (Li^+^) conductors on lithium surfaces, such as LiN_x_O_y_ and Li_3_N, which facilitate even Li^+^ deposition. Thus, it significantly improves the performance of EEIs.^[^ ^12^, ^13^ ^]^ Based on this, combining the anti‐high voltage characteristics of SN with the excellent film‐forming capability of LiNO_3_ is expected to achieve stable operation of LMBs under extreme conditions. However, LiNO_3_ has limited solubility in ester and nitrile electrolytes, especially in carbonate electrolytes, where the solubility is only about 800ppm.^[^ ^14^, ^15^ ^]^ Therefore, extensive studies have been conducted to improve the dissolution of LiNO_3_ in ester electrolytes by introducing solvents/additives with high donor numbers,^[^ ^16^ ^]^ high dielectric constants,^[^ ^17^ ^]^ and even the addition of trace amounts of metal fluorides.^[^ ^18^ ^]^ Although these strategies effectively increase the solubility of LiNO_3_, they also result in solvent consumption during EEI formation. Therefore, there is an urgent need to develop new strategies that broaden the application of LiNO_3_ in high‐voltage electrolytes to improve the cycling performance of LMBs.

**Figure 1:**
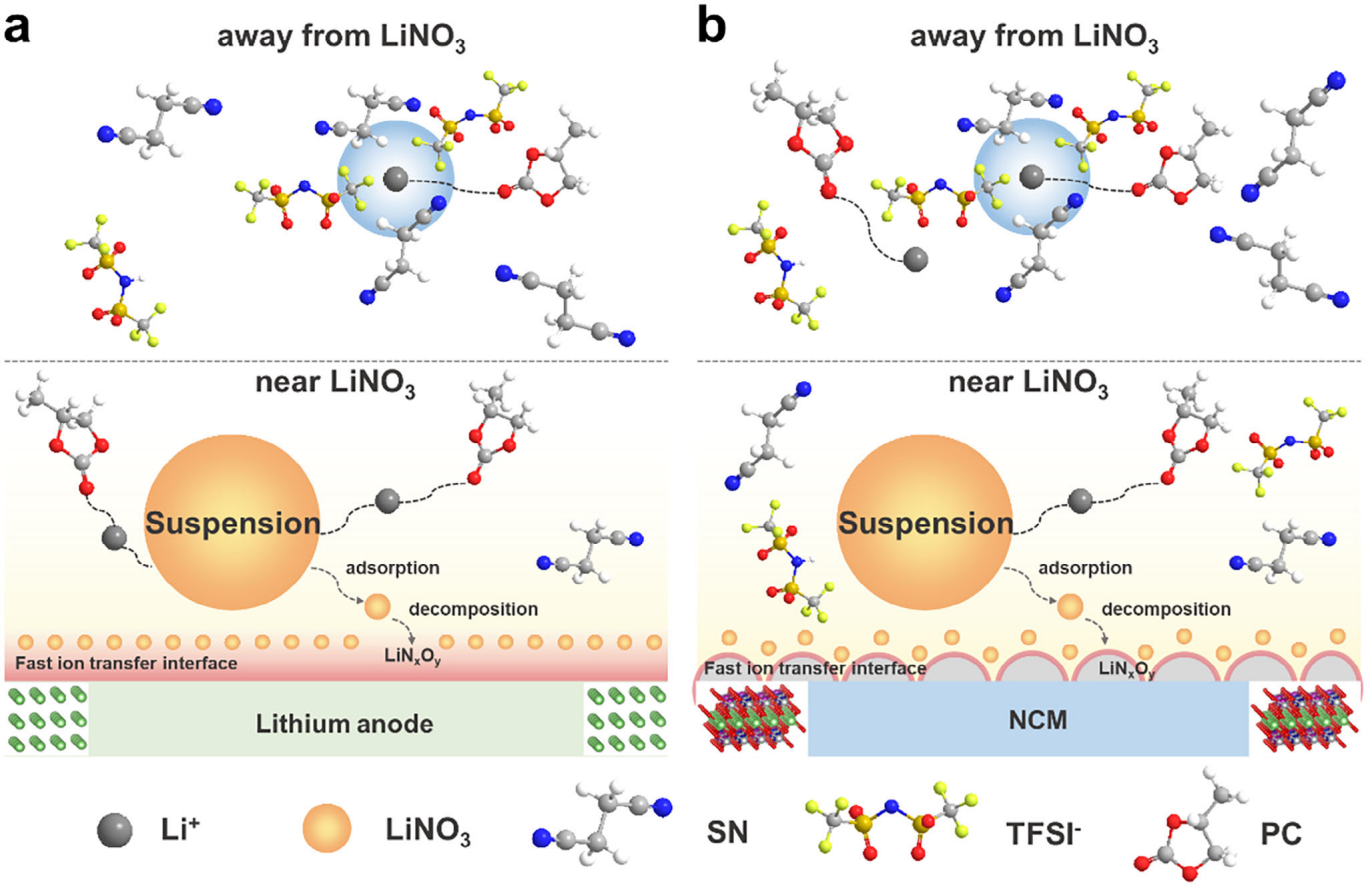
Mechanism diagram of LN‐LTPCS to improve the performance of Li‐metal batteries. a) Mechanism diagram of suspension electrolyte optimization for SEI and b) CEI.

**Figure 2:**
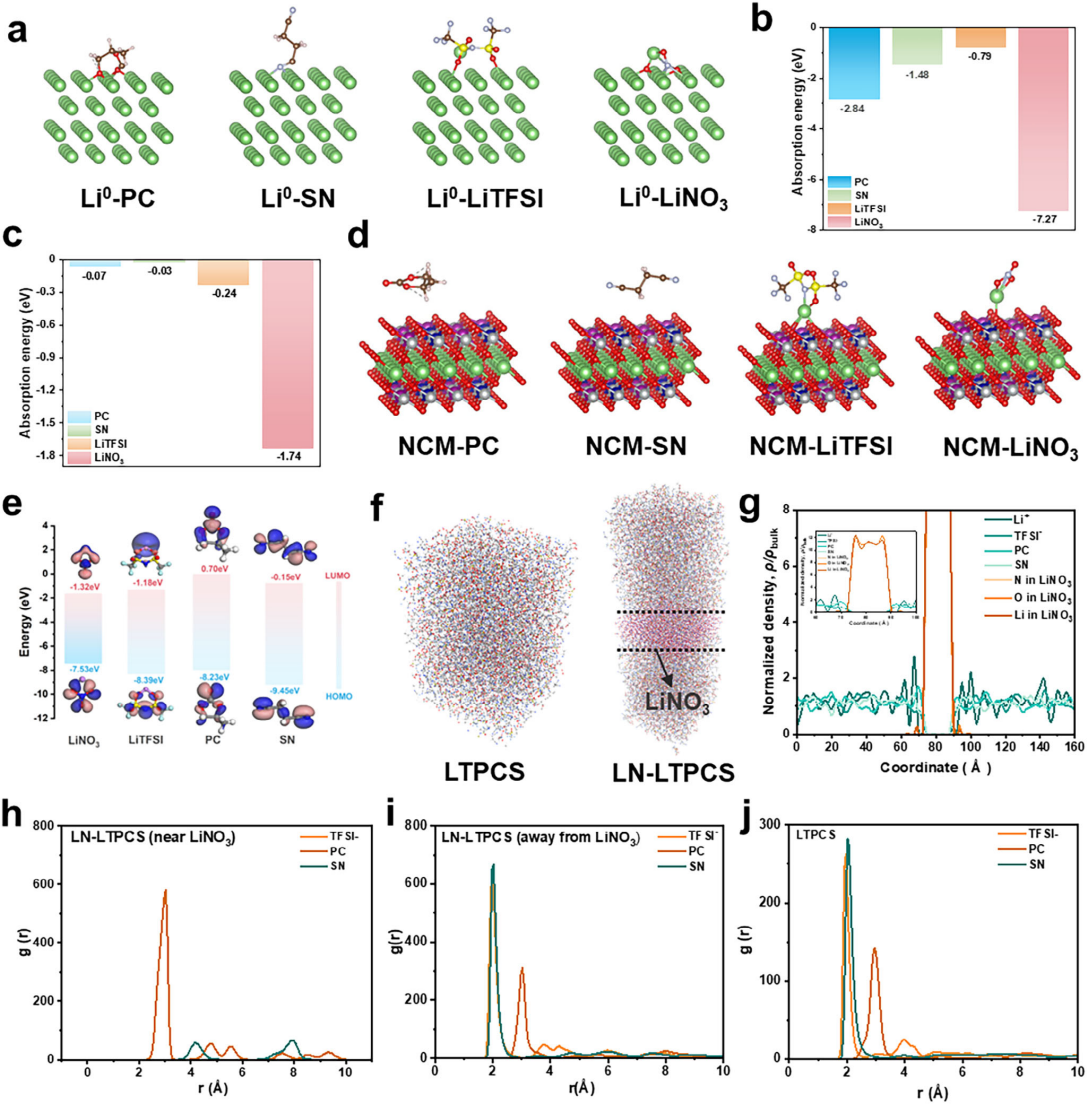
a) The adsorption structures of PC, SN, LiTFSI, LiNO_3_ on Li (110) metal anode and b) their adsorption energy (E_ads_). c) The E_ads_ and d) adsorption structures of PC, SN, LiTFSI, and LiNO_3_ on NCM523 (003) cathode. e) HOMO, LUMO energy levels of LiNO_3_, LiTFSI, PC, SN. f) Snapshots of MD simulations of molecular species (Li^+^, TFSI^−^, PC, and SN) for LTPCS and LN‐LTPCS. g) Density profiles view of LN‐LTPCS with the LiNO_3_ slab, The ρ and ρ_bulk_ represent density and bulk density of the specified species. h) RDFs for Li^+^ solvation shell of LN‐LTPCS near the LiNO_3_ slab (<5 Å), The g(r) represents the radial distribution. i) RDFs for Li^+^ solvation shell of LN‐LTPCS away from the LiNO_3_ slab (5 Å from the furthest end of the box). j) RDFs for the Li^+^ solvation shell of LTPCS.

Here, we propose a novel suspension electrolyte design by introducing LiNO_3_ in the ether‐free electrolyte composed of propylene carbonate (PC)/SN as mixed solvents along with lithium bis(trifluoromethanesulphonyl)imide (LiTFSI) as salt, referred to as LN‐LTPCS. PC and SN are chosen due to their low melting point, high boiling point, and high flash point, which help to improve the high temperature performance of batteries.^[^ ^19^ ^]^ This innovative electrolyte enables LMBs to achieve excellent cycling stability at wide temperatures (‐10∼60 °C) and high voltage (4.5 V). The mechanism of the designed suspension electrolyte is illustrated in **Figure** **1**a. Through theoretical calculations and experimental analyses, several key functions of LiNO_3_ have been validated: (1) LiNO_3_ changes the solvation environment of Li^+^ in the surrounding liquid electrolyte through its coordination with SN and TFSI^−^ by electrostatic interaction, which subtly improves the compatibility between the electrolyte and the electrode. (2) LiNO_3_ preferentially adsorbs on the electrode surface (**Figure** **2**a–d), and has the lowest unoccupied molecular orbital (LUMO) energy (Figure 2e), which means that LiNO_3_ can be decomposed preferentially to form EEIs during battery operation. The formed stable EEIs help fast ion transport, accelerating the overall reaction rate.^[^ ^20^ ^]^ Therefore, the designed electrolyte system makes Li||LiNi_0.5_Co_0.2_M_0.3_ (NCM523) battery run stably for 100 cycles with a high capacity retention of 90.05% at a high temperature of 60 °C. Moreover, the electrolyte has excellent electrochemical stability at low temperatures (‐10 °C) and a high cut‐off voltage of 4.5 V.

## 2 Results and Discussion

### 2.1 Solvation Structures

In order to elucidate the effect of the Li^+^ solvent environment on EEI formation in LTPCS and LN‐LTPCS electrolytes, we performed molecular dynamics (MD) simulations and Raman tests. Figure 2a–d show that LiNO_3_ has lower adsorption energy compared to other electrolyte compositions, implying the preferential adsorption of LiNO_3_ on the lithium metal and NCM523 cathode surface. This preferential interaction can modulate the electrode surface and form a stable EEI. Figure 2f presents snapshots of the LTPCS and LN‐LTPCS electrolytes, while the normalized density distribution for the LN‐LTPCS electrolyte is illustrated in Figure 2g. The results indicate that the density peak of Li^+^ is considerably higher than that of other electrolyte components. This phenomenon can be attributed to the strong adsorption of Li^+^ and LiNO_3_, which leads to a distinct solvation structure of Li^+^ around the LiNO_3_ slab in LN‐LTPCS, contrasting with the solvation structure observed in the LTPCS system without the LiNO_3_ slab.

Radial distribution functions (RDFs) were utilized to analyze the solvation structures of the electrolytes both at 5 Å from the LiNO_3_ slab in LN‐LTPCS (Figure 2h) and further from the LiNO_3_ interface (Figure 2i), as well as in LTPCS (Figure 2j). The analysis reveals that the presence of the LiNO_3_ slab in the LN‐LTPCS system significantly alters the Li⁺ solvation environment compared to the LTPCS electrolyte system. Notably, near the LiNO_3_ slab, Li⁺ is predominantly solvated by PC, whereas the concentration of SN decreases substantially, and coordination with TFSI^⁻^ at the LiNO_3_ slab is absent. Conversely, in regions further away from the LiNO_3_ slab, the presence of SN and TFSI^⁻^ surrounding Li⁺ increases, suggesting that the LiNO_3_ slab repels these components such as SN and TFSI^⁻^. Moreover, Raman was used to deeply study the effect of LiNO_3_ on the solvated structure. First, the Raman spectrum of Figure S3a (Supporting information) shows a significant shift of the NO_3_^−^ stretching vibrational peak of LiNO_3_, confirming the participation of trace NO_3_^−^ in the solvation of Li^+^. Second, Figure S3b,c (Supporting information) reveal the Li^+^‐solvent‐anion interactions and the synergistic evolution of the solvated structure. In the suspended electrolyte LN‐LTPCS, the Li^+^‐TFSI^−^ coordination is significantly diminished, indicating that suspended LiNO_3_ repels TFSI^−^, detaching from the main solvation shell. Moreover, the coordination of solvated SN and free SN is also significantly reduced due to the presence of suspended LiNO_3_ particles, suggesting that the suspended LiNO_3_ particles inhibit the solvation of SN to some extent. This is in agreement with the simulation data.

The effect of LiNO_3_ plates on the dissolved environment enhances the compatibility of the electrolyte‐electrode interface. Previous analyses have shown that LiNO_3_ has a stronger adsorption effect on lithium metal (Figure 2b,c). Due to this strong interaction, lithium metal tends to adsorb dissolved Li^+^ rather than SN and TFSI^−^. This interaction mitigates the corrosive effect of SN on the lithium metal anode and the side reactions at the electrolyte‐anode interface, thereby improving the stability of the anode and the overall cycling performance of LMBs. The results of the scanning electron microscope (SEM) further confirmed these findings. Here the bare lithium foils were soaked into LTPCS and LN‐LTPCS for one week, respectively, and the topography was observed using SEM. The results show that the surface of the samples containing LiNO_3_ is smooth and free of corrosion pits (Figure S4a,b, Supporting Information), whereas the control group shows severe corrosion due to the strong corrosion coming from SN. It is further verified that the presence of LiNO_3_ can effectively inhibit the corrosion of SN. For the cathode, the adsorption of LiNO_3_ on the NCM cathode was relatively weak, resulting in higher SN and TFSI^−^ coordination at the cathode. The increased concentration of SN and TFSI^−^ contributes to the stabilization of the CEI.

### 2.2 Battery High‐Voltage Performance

**Figure 3:**
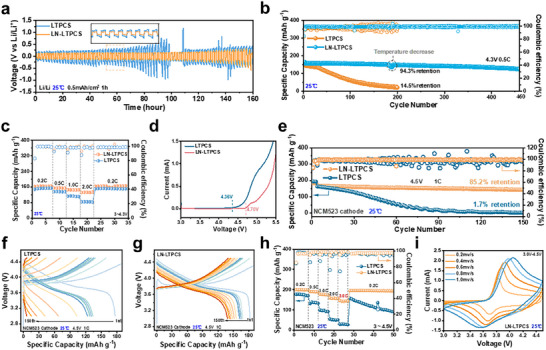
Cycling performances of a) Li symmetrical battery at 0.5 mA cm^−2^ with a capacity of 0.5mAh cm^−2^. b) Li||NCM523 battery long cycle test at 3∼4.3 V. c) Li||NCM523 battery multiplication performance at 4.3 V. d) LSV curves for Li||LTPCS||NCM523 and Li||LN‐LTPCS||NCM523. e) Long cycle test of Li/NCM523 cell at 3∼4.5 V. f) Specific capacity voltage curve for Li||LTPCS||NCM523 at 3∼4.5 V. g) Specific capacity voltage curve in the voltage range of 3∼4.5 V for Li||LN‐LTPCS||NCM523. h) Rate performance of Li||NCM523 cell at 4.5 V. i) CV test in the voltage range of 3∼4.5 V for different sweep speeds of Li||LN‐LTPCS||NCM523 cell.

To verify the effect of electrolytes on the performance of lithium metal batteries, lithium symmetric cells as well as Li||NCM523 cells were assembled and the effects of solvent type and additive content on the battery were tested. Figure S1 (Supporting information) further shows the Li||NCM523 battery with electrolyte without PC (LTS) has nearly no capacity at room temperature. Further modulating the additive content, Figures S5 and S6 (Supporting information) show that the battery presents the highest specific capacity when the LiNO_3_ content is 4%. Based on this, the electrolyte with 4% LiNO_3_ addition (LN‐LTPCS) was subsequently further characterized and analyzed, and the mechanism of LiNO_3_ effect on the battery performance was investigated. As shown in **Figure** **3**a, Li||Li with LN‐LTPCS showed a more stable polarisation voltage compared with LTPCS. Figure 3b and Figure S7 (Supporting information) show Li||NCM523 with LN‐LTPCS has good cycling stability with 94.3% capacity retention after 200 cycles and over 99.2% Coulombic efficiency at 4.3 V cut‐off voltage; the capacity retention is still maintained at around 80% after 460 cycles. In contrast, the LTPCS showed significant capacity degradation after 40 cycles and only 14.5% capacity retention after 200 cycles. This is related to the large polarisation that occurs within the LTPCS battery system (Figure S8, Supporting information). Figure 3c shows that the charge/discharge tests of Li||LN‐LTPCS||NCM523 batteries at different rates of 0.2, 0.5, 1.0, 2.0, and 0.2 C resulted in the specific capacities of 162.99, 155.74, 145.2, and 132.65 mAh g^−1^, and back to 0.2 C resulted the capacity of 164.91 mAh g^−1^, indicating that the LN‐LTPCS has good rate performance.

Linear cyclic voltammetry (LSV) was used to evaluate the oxidation stability of LN‐LTPCS. Figure 3d shows that LN‐LTPCS can maintain stability at a high voltage of 4.5 V. Therefore, even at a high‐voltage of 4.5 V, the cycle stability and rate performance of Li||NCM523 with LN‐LTPCS is still better than that of LTPCS. Specifically, the Li||NCM523 battery system with LN‐LTPCS possesses an initial specific capacity of 184 mAh g^−1^ (Figure 3e), remains a reversible capacity of 143.01 mAh g^−1^ after 150 cycles with a capacity retention rate of 85.2% at 1.0 C (Figure S9, Supporting information). In contrast, the capacity retention of the LTPCS system is only 1.7% after 150 cycles. The voltage‐capacity curves of Figure 3f,g further confirmed the internal polarisation of the Li||LTPCS||NCM523 cells due to the severe side reaction, which led to the capacity loss. The rate performance of Li||LN‐LTPCS||NCM523 cell was tested at 0.2, 0.5, 1.0, 2.0, and 3.0 C, and their specific capacities were 199.2, 189.0, 173.8, 156.3, and 142.1 mAh g^−1^, respectively (Figure 3h), which confirmed they have considerable rate performance at high. The cyclic voltammetry (CV) result of the Li||NCM523 cell shows that the LN‐LTPCS electrolyte has better reversibility (Figure 3i; Figure S10 and S11, Supporting information). Besides, Figure S12 (Supporting information) shows that LN‐LTPCS electrolyte has a relatively high diffusion coefficient. The results showed that the presence of LiNO_3_ largely stabilized the interfacial layer; furthermore, it was confirmed that the LN‐LTPCS had fast ion transport, which could be attributed to the formation of a fast ion interfacial by LiNO_3_.^[^ ^21^ ^]^

**Figure 4:**
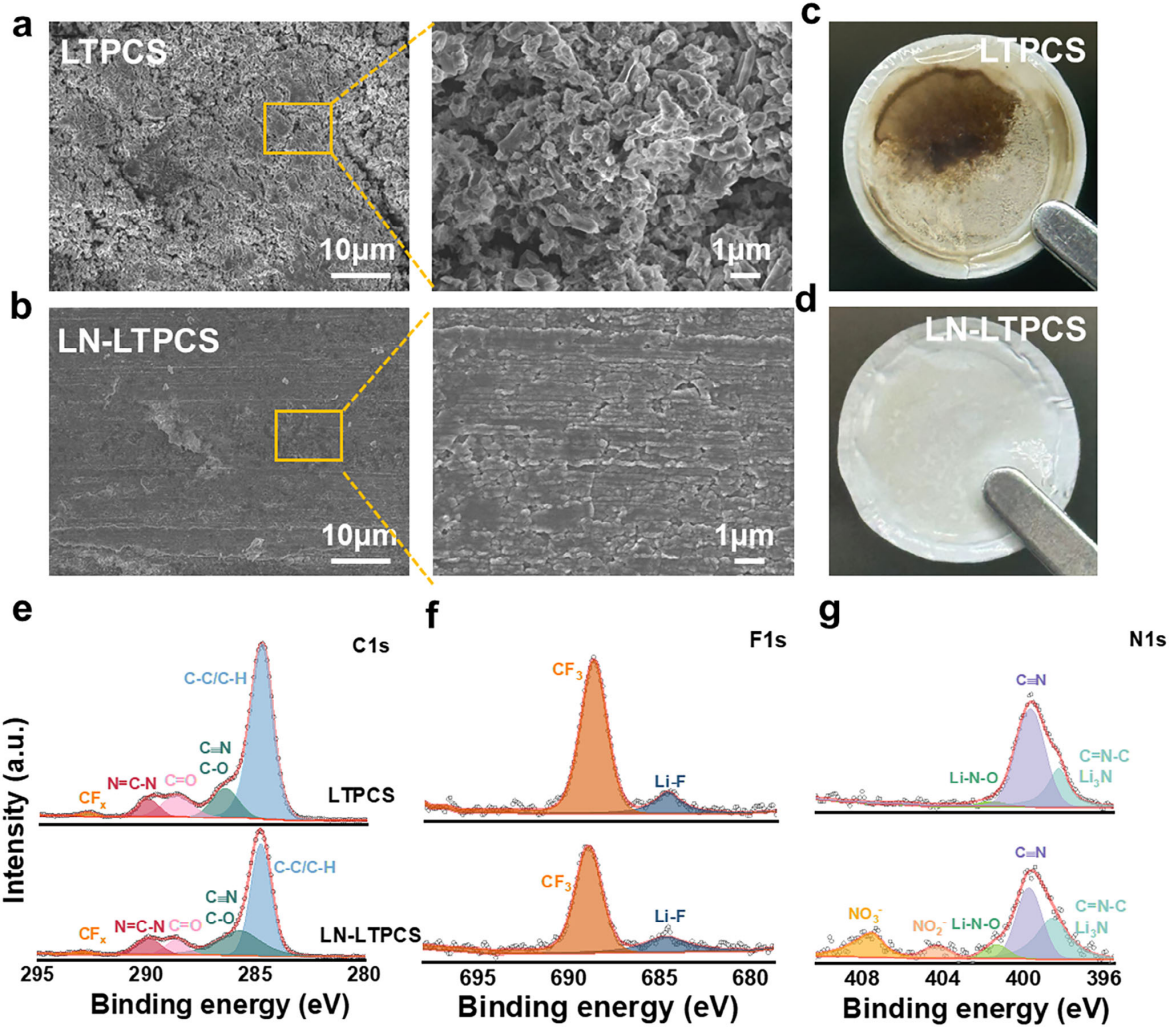
a) The surface morphology of lithium metal after LTPCS cycling at different magnifications. b) The morphology of lithium metal after LN‐LTPCS cycling at different magnifications. c, d) Separator state after 150 cycles of LTPCS and LN‐LTPCS under 4.5 V cut‐off voltage. e, f, g) are the C1s, N1s, and F1s spectra of the Li anode interface after cycling 20 cycles of Li||LTPCS||NCM523 and Li||LN‐LTPCS||NCM523 at high‐voltage 4.5 V.

To further elucidate the effect of the electrolyte system on the interfacial chemistry, SEM and X‐ray photoelectron spectroscopy (XPS) characterization were performed to characterize the anode and cathode after cycling at 4.5 V. The lithium deposition morphology of Li||NCM523 cell was characterized after 150 cycles. Compared with the loose and porous lithium deposits with more surface cracks in the LTPCS (**Figure** **4**a), the lithium deposits in the LN‐LTPCS are tightly packed with a flat surface (Figure 4b). This may be because lithium metal susceptible reacted with SN and PC to form inhomogeneous and thick SEI as well as uncontrollable lithium dendrite growth, which leads to poor cycling performance and Coulombic efficiency of the cells. The separator also reflects the violent side reactions occurring inside the LTPCS‐based cell (Figure 4c,d). XPS was used to further analyze Li surface composition after 20 cycles. As shown in Figure 4e,f, the Li surface of the LTPCS‐based cell shows lower contents of CF_3_ in the C1s and F1s spectrum,^[^ ^22^, ^23^, ^24^ ^]^ this may be due to the decomposition of a few anions near the Li surface. In addition, N1s spectra (Figure 4g) show the presence of NO_2_^−^ (404.3 eV), and NO_3_^−^ (407.6 eV) on the Li surface with LN‐LTPCS,^[^ ^25^ ^]^ which is absent in the LTPCS. It is hypothesized that in LN‐LTPCS, the preferential adsorption of the LiNO_3_ layer on the electrode interface protects the electrode, repelling corrosive SN through the adsorption of PC‐solvated Li^+^ and reducing the decomposition of the solvent.^[^ ^26^, ^27^ ^]^ This process induces the generation of SEIs containing inorganic LiN_x_O_y_ with fast ion transport properties, improving the lithium‐ion migration rate.^[^ ^28^, ^29^, ^30^ ^]^ Therefore, Li||NCM523 cells with LN‐LTPCS show a low interface impedance (Figure S13, Supporting information),^[^ ^31^ ^]^ and achieve superior durability and higher Coulombic efficiency.

**Figure 5:**
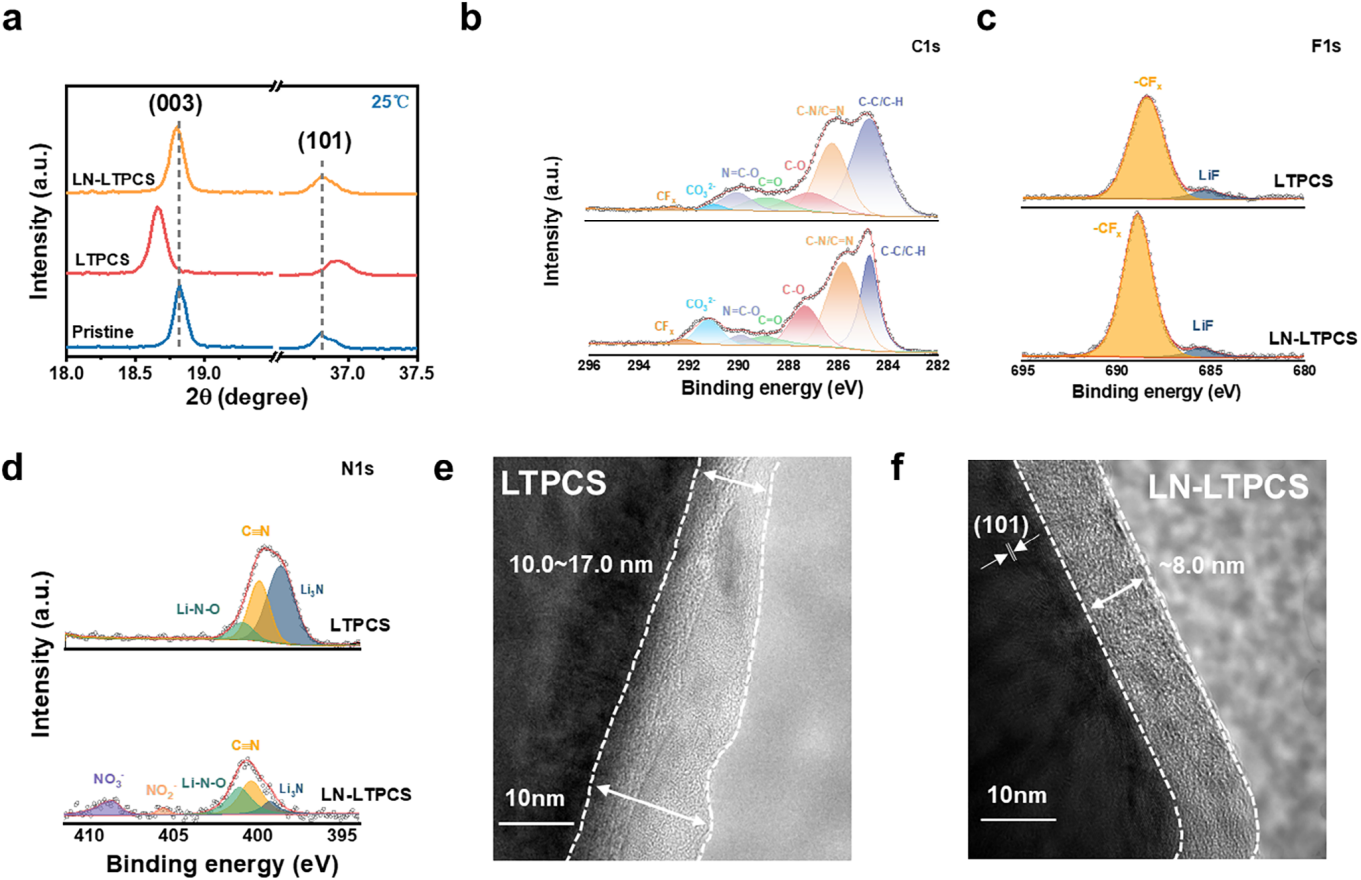
a) XRD of NCM523 after high voltage cycling. b, c, d) C1s, N1s, and F1s spectra of the NCM523 cathodic interface after 20 cycles of cycling at 4.5 V for Li||LTPCS||NCM523 versus Li||LN‐LTPCS||NCM523 at high power, respectively. e, f) TEM images of the positive electrode of Li||NCM523 after 60 cycles of cycling at 3∼4.5 V, respectively.

The cycling stability of the battery not only depends on the SEI stability but also on the CEI. X‐ray diffraction (XRD) tests were performed on the cathode after high cut‐off voltage cycling (Figure S14, Supporting information). The results show that the corresponding peaks between the (003) and (101) crystal faces of NCM523 are less displaced after cycling in the LN‐LTPCS (**Figure** **5**a), indicating minimal structural changes during Li^+^ intercalation/ deintercalation.^[^ ^32^, ^33^ ^]^ This is because the LiNO_3_‐formed interface protects the electrode.^[^ ^34^ ^]^ To further explore the effect of LN‐LTPCS on the cathode, XPS was performed on NCM523 after 20 cycles. As shown in Figure 5b,c, LTPCS shows a lower CF_3_ fraction in C1s and F1s spectra (292.4, 692.0 eV) from TFSI^−^ on the cathodic surface than in LN‐LTPCS.^[^ ^31^ ^]^ This is because there is relatively less LiNO_3_ adsorbed on the cathode side compared to the anode side, the anion repulsion is relatively weak, and the TFSI^−^ can decompose on the cathode side to form CEI. The ‐CF_3_ content of LTPCS>LN‐LTPCS on the anode side and the opposite trend on the cathode side provide evidence for the theory that the LiNO_3_ layer dynamically regulates the electrode‐electrolyte interface. Besides, the CEI of LN‐LTPCS (Figure 5d) also has the presence of NO_3_^−^ (408.8 eV), NO_2_^−^ (405.6 eV), and less C≡N (399.6 eV), suggesting that the preferential decomposition of LiNO_3_ and participates in the formation of a fast ion‐transporting CEI layer. Such a CEI not only inhibits the dissolution of interfacial CEI by the polar solvent,^[^ ^34^, ^35^ ^]^ but also blocks the continuous reaction between the electrolyte. In contrast, there is more decomposition of organic solvents on the CEI of LTPCS, confirming irreversible electrochemical decomposition occurs inside the cell.^[^ ^36^, ^37^ ^]^ TEM confirmed the optimization of CEI by LiNO_3_ additive. Figure 5e shows the CEI in LTPCS was more inhomogeneous and with a thickness was about 10.7∼17 nm, much thicker than that of LN‐LTPCS, which was about 8 nm and homogeneous (Figure 5f). The results show that the LiNO_3_ induces the formation of dense and thin CEI at the cathode interface. It not only prevents the cathode material from directly contacting the electrolyte to reduce the side reaction but is also used as a lithium source. Combined with high‐voltage resistant SN, synergistically improves the high‐voltage stability of the battery. Therefore, LN‐LTPCS both improves the compatibility with the lithium anode and the cathode.

### 2.3 Battery Performance at Extreme Temperature

**Figure 6:**
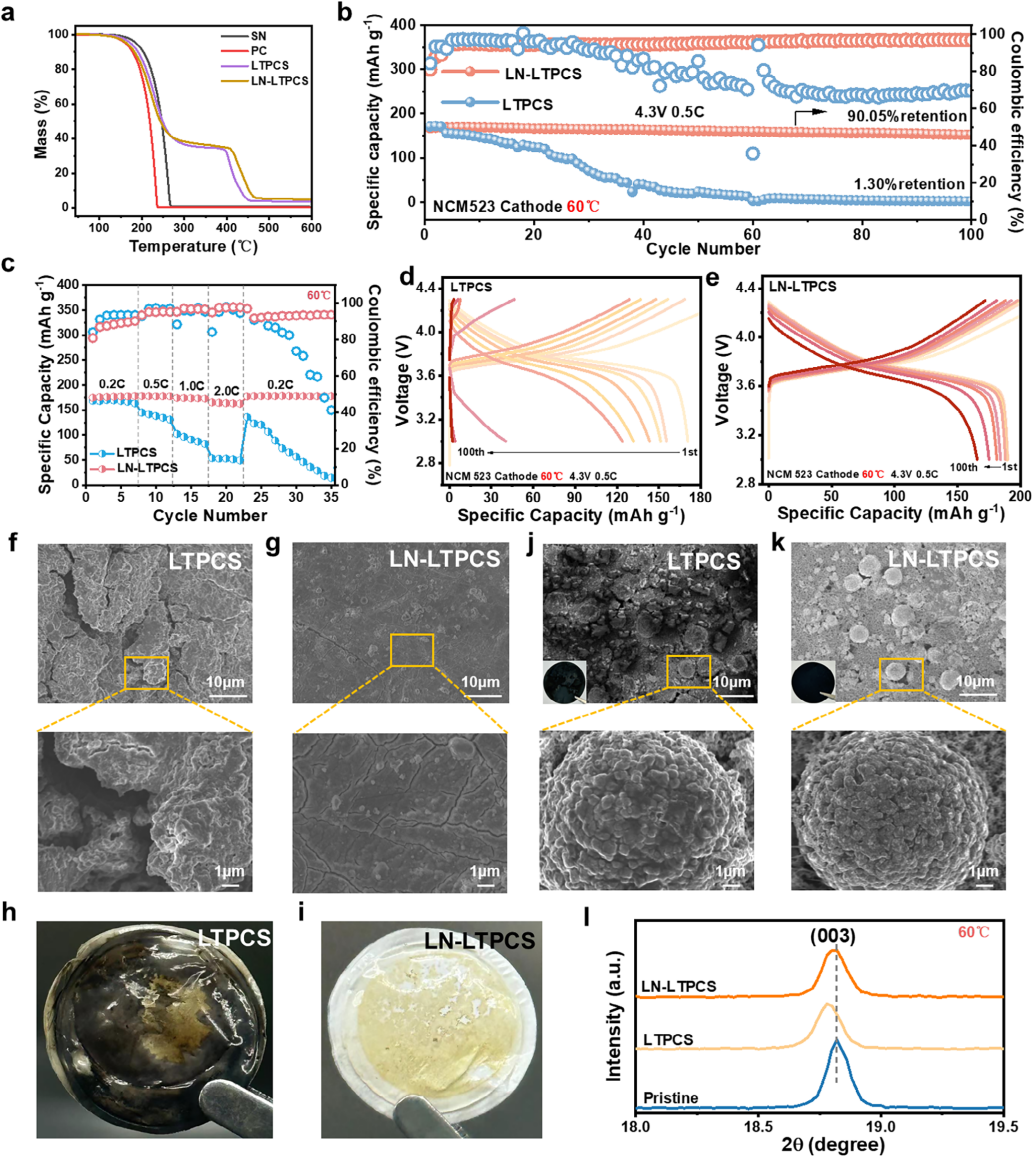
a) Thermal decomposition curves of electrolyte components. b) Electrochemical performance of Li||NCM523 full cell at 3∼4.3 V at (60 °C). c) Rate performance Li||NCM523 battery at 3∼4.3 V at 60 °C. d) Specific capacity voltage curve of Li||LTPCS||NCM523. e) Specific capacity voltage curve of Li||LN‐LTPCS||NCM52. f) Lithium metal surface morphology after 100 cycles of Li||LTPCS||NCM523 at 60 °C with different magnifications. g) Lithium metal surface morphology after 100 cycles of Li||LN‐LTPCS||NCM523 at 60 °C at different magnifications. h, i) Separator state of Li||LTPCS||NCM523 and Li||LN‐LTPCS||NCM523 cells after 100 cycles at 3∼4.3 V at 60 °C. j, k) Surface morphology of NCM523 after 100 cycles at 4.3 V voltage for Li||LTPCS||NCM523 and Li||LN‐LTPCS||NCM523 batteries at 60 °C. l) XRD of the cathode after cycling under different conditions.

**Figure** **6**a shows both LTPCS and LN‐LTPCS had better thermal stability, with decomposition onsets at about 150 °C with about 95% mass lost above 450 °C. The thermal performance of the LN‐LTPCS with LiNO_3_ was slightly higher than that of the LTPCS system, this excellent thermal stability is sufficient for use in LMBs. It was found the Li||LN‐LTPCS||NCM523 battery exhibits excellent performance at high temperatures. The LN‐LTPCS system has a high initial specific capacity of 184 mAh g^−1^ and 90% capacity retention after 100 cycles at high temperatures, whereas the LTPCS system has only 1.3% capacity retention (Figure 6b). Moreover, LN‐LTPCS electrolyte makes Li||NCM523 battery shows good repeatability (Figure S15, Supporting information), and better rate performance(Figure 6c). Electrolyte depletion is one of the main reasons for the degradation of cell performance, especially under extreme conditions.^[^ ^38^ ^]^ Compared to the LTPCS system, the Li||NCM523 cells with LN‐LTPCS have better electrochemical stability at high temperatures, which is due to the formation of a more stable EEI induced by LiNO_3_, reduced internal reaction in the battery (Figure 6d,e). SEM showed that compared to the lithium surface morphology of LTPCS, which is rough and porous (Figure 6f), the surface of LN‐LTPCS is smooth and compact (Figure 6g). As LiNO_3_ particles are adsorbed and deposited on the electrode surface, they not only form a protective layer, but their strong repulsion with SN will drive away SN molecules and inhibit interfacial side reactions. When the internal temperature of the battery reaches 50 °C, LiNO_3_ in the electrolyte decomposes and induces the formation of dense SEI, slowing down the consumption of solvent.^[^ ^39^ ^]^ Moreover, the clean separator after high‐temperature cycling (Figure 6h,i) further reflects the milder internal reaction in the LN‐LTPCS system.

In fact, cathode materials also face interfacial instability challenges,^[^ ^32^ ^]^ especially in high temperatures. The surface morphology of NCM523 after cycling under high‐temperature conditions was investigated by SEM. Figure 6j,k and Figure S16 (Supporting information) show that cracks appeared on the anode surface of the LTPCS system after high‐temperature cycling, while the anode surface of LN‐LTPCS system was more intact after cycling. Besides, irreversible phase transitions and volume changes induced by mixed cations are the main reasons for the thermal degradation and cycling stability of batteries.^[^ ^36^, ^40^, ^41^, ^42^, ^43^, ^44^ ^]^ High temperatures accelerate this process because due to rapid insertion/desertion of Li^+^and interface reaction.^[^ ^36^ ^]^ XRD showed (Figure 6l) confirms the above idea that the crystalline surface of NCM523 (003) of LTPCS was shifted after high‐temperature cycling, while LN‐LTPCS did not undergo any significant change. In conclusion, the preferential adsorption of LiNO_3_ particles separates the electrolyte from the electrode, and the LiNO_3_‐formed layer inhibits the penetration of the electrolyte and the constant side reactions, which reduces the interfacial reaction (Figure S17, Supporting information). On the other hand, the LiNO_3_ plate will influence the coordination of Li^+^ solvation and can cause anions to accumulate at the cathode by repelling them, forming an anion‐rich CEI, which effectively reduces the continuous consumption of organic solvent. At the same time, LiNO_3_ also plays the role of Li‐ion supplementation, hindering lithium‐nickel mixing and crack expansion,^[^ ^42^, ^45^ ^]^ The results show that LiNO_3_ is effective in inhibiting the phase transition and microcracking of NCM523 during cycling even under high‐temperature conditions. In addition to keeping stable at high temperatures, Li||NCM523 with LN‐LTPCS electrolyte remains stable to run for 100 cycles without a significant increase in polarization voltage at ‐10 °C (Figures S18 and S19, Supporting information).

## 3 Conclusion

In this work, we propose a suspension electrolyte design to obtain LMBs adapted to extreme environments. A detailed study based on a LiNO_3_ suspension electrolyte was carried out. Computational and experimental characterization demonstrates that LiNO_3_ particles are in the suspended state, affecting electrolyte solvation structure and interfacial composition by the strong interaction with the electrolyte and the strong adsorption with the interface. On one side, LiNO_3_ particles preferentially adsorb on lithium anode to reduce corrosion of anode coming from SN and promote an anion‐rich cathode interface form via exclusionary effect. At the same time, LiNO_3_ undergoes self‐decomposition and induces the formation of a stable LiN_x_O_y_‐rich EEI. The stable EEI favors rapid ion transport and effectively inhibits the electrode/electrolyte irreversible side reaction. The designed electrolyte system has excellent electrochemical stability at a high‐voltage of 4.5 V and makes the Li||NCM523 battery run stably at wide temperatures of ‐10∼60 °C, e.g., Li||NCM523 battery run for 100 cycles with high capacity retention of 90.05% at a high temperature of 60 °C. This study demonstrates that rational electrolyte design enables the construction of stable electrode‐electrolyte interphases, and enhances cycling resilience across wide temperature ranges.

## Conflict of Interest

The authors declare no conflict of interest.

## Author Contributions

W.Z. acquired and analyzed most of the experimental data and wrote the manuscript under the guidance of Y.L. Z.Z. and H.Z. acquired and analyzed the simulated data. X.L. offered expertise in the computational simulation. All data were analyzed under the guidance of Y.L. and Z.R., and the results were further cross‐checked. Y.L. and Z.R. conceived the work and were in charge of overall direction and funding support. All the authors reviewed and edited the manuscript.

## Supporting information



Supporting Information

## Data Availability

The data that support the findings of this study are available from the corresponding author upon reasonable request.
