# Supplementary material for: Suspended Lithium Nitrate‐Based Electrolytes: Electrostatic Interactions for Mutually Rewarding Interface Optimization Strategies
Source: Adv Sci (Weinh). 2025 Mar 20;12(18):2416656. doi: 10.1002/advs.202416656 (PMC12079540; doi:10.1002/advs.202416656)
Supplement: Supplementary file 1 — Supporting Information [file ADVS-12-2416656-s001.docx]

**Suspended lithium nitrate-based electrolytes: electrostatic interactions for mutually rewarding interface optimization strategies**

*Wenjing Zhang, Zhenguo Zhang, Hongtao Zhang, Yang Luo^*^, Xinjian Liu^*^, Zhonghao Rao^*^*

W. Zhang, Z. Zhang, H. Zhang, Y. Luo, X. Liu, Z. Rao

Hebei Engineering Research Center of Advanced Energy Storage Technology and Equipment, School of Energy and Environmental Engineering, Hebei University of Technology, Tianjin, 300401, China.

W. Zhang, Z. Zhang, H. Zhang, Y. Luo, X. Liu, Z. Rao

Hebei Key Laboratory of Thermal Science and Energy Clean Utilization, School of Energy and Environmental Engineering, Hebei University of Technology, Tianjin, 300401, China.

W. Zhang, Z. Rao

School of Materials Science and Engineering, Hebei University of Technology, Tianjin 300401, China.

W. Zhang, Z. Zhang, Y. Luo, X. Liu, Z. Rao

State Key Laboratory of Intelligent Power Distribution Equipment and System, Hebei University of Technology, Tianjin, 300401, China.

E-mail: Yang Luo([luoyang@hebut.edu.cn](mailto:luoyang@hebut.edu.cn)), Xinjian Liu([liuxinjian@hebut.edu.cn](mailto:liuxinjian@hebut.edu.cn)), Zhonghao Rao([raozhonghao@hebut.edu.cn](mailto:raozhonghao@hebut.edu.cn));

**Methods and materials**

Materials: Lithium metal anode(700 μm) was purchased from (China Energy lithium Co, Ltd), diameter 16 mm. Separator Celgard 2325 is cut into round pieces with a diameter of 19 mm, used for assembling button batteries. LiNi_0.5_Co_0.2_Mn_0.3_O_2_ cathode materials (NCM523), super p, PVDF was fully dried under vacuum before use, and prepared following NCM523: super p: PVDF mass ratio of 8:1:1 to prepare a slurry, with magnetic stirring at 800 r/rotation speed for 4 h, and then coated on Al foil at a thickness of 80 μm, and fully dried under vacuum at 60 ℃, and cut into electrode discs with a diameter of 14 mm. Lithium nitrate (LiNO_3_, damas-beta, 99%) was dried at 100 °C before use. Lithium bis(trifluorosulfonyl)imide (LiTFSI, Shangfluoro, analytically pure (AR)), propylene carbonate (PC, Clorox, analytically pure (AR)), and Succinonitrile (SN, Aladdin, 99%). The above materials should be fully dried at 60 °C before use and stored in a glove box filled with argon gas (H_2_O<0.1 ppm, O_2_<0.3 ppm).

Electrolyte preparation: Electrolyte preparation: The eutectic electrolyte LiTFSI:SN=1 mol:1 mol was prepared in a glove box and designated as LTS. Eutectic LTS (LiTFSI:SN=1 mol:1 mol) electrolyte has a high viscosity and the migration of Li^+^ is slow, which resulted in the failure of the cell to work (Fig. S1). Therefore, PC was added as the dilution solvent of the LTPC electrolyte, and a eutectic electrolyte with a molar ratio of 1:3:3 was prepared with LiTFSI: PC: SN as the comparison electrolyte (referred to as LTPCS); the experimental electrolyte was made by adding 4 wt% of lithium nitrate on top of LTPCS, and stirred at 1000 r/s until a homogeneous milky white color was formed, which indicated that LiNO_3_ particles were fully broken and evenly dispersed, sufficiently broken and uniformly dispersed, which is recorded as LN-LTPCS.The settling time (24 h) of the multi-scale particles in this experiment still qualitatively reflects the suspension stability. Moreover, after one day of settling, a uniform milky white color can be formed by simply shaking for 10 times, which proves that the LiNO3 particles have not irreversibly agglomerated and the levitated electrolyte has good re-dispersibility (Figure S2).

Electrochemical tests: Button cells were assembled in an argon-filled (H_2_O<0.1 ppm, O_2_<0.3 ppm) glove box. Electrode shells (2016R) and spacers (0.5 mm) were purchased from (Guangdong Canrd New Energy Technology Co, Ltd). Celgard 2325 separator with a diameter of 19 mm was used, and 80 μl of electrolyte was added dropwise to the full cell, and 50 μl to the symmetric cell. The assembled batteries were charged and discharged on the LANBTS Battery Tester (BT-3018A, Rambo) under the ambient conditions of constant 25°C and constant 60°C, respectively. The voltage ranges from 3~4.3 V (vs. Li/Li^+^) was activated by cycling 3 times at 0.2 C for 3 cycles for activation, and the rest of the cycles at 0.5 C for long cycle charge/discharge test; assembled full battery, and battery multiplication performance at different multiplication conditions of 0.2, 0.5, 1.0, 2.0, and 0.2 C (5 cycles each). Long cycle charge/discharge test at 1.0 C after activating the battery by cycling at 0.2 C for 3 times at 25 ℃ under the high voltage range of 3~4.5 V. Assembling the whole battery, the multiplicity performance of the battery to withstand high voltage under different multiplicity conditions of 0.2, 0.5, 1.0, 2.0, 3.0, 0.2 C (5 cycles each). Cyclic voltammetry was performed using a DH7000 electrochemical workstation, and linear cyclic voltammetry and electrochemical impedance (200 kHz ~100 mHz) were performed using a BioLogic SP-300 electrochemical workstation.

Characterization methods: Thermogravimetric analysis was carried out using a NETZSCH (STA 449 F5 Jupiter) to test the thermal stability of the electrolyte. The test was carried out under nitrogen atmosphere in the temperature range of 40~600 °C with a ramp rate of 10 °C/min. The morphology of the electrode surface after cycling was analysed using a scanning electron microscope (JSM 7610F, Japan). Transmission electron microscopy (JEM 2100F, Japan) was used to observe the surface of the positive electrode after high-temperature cycling (after drying the treated electrode in a glove box using DME washing). The post-cycling electrode surface was analyzed using an X-ray photoelectron spectrometer (ESCALAB 250xi, Thermo Fisher) using monochromatic Al K α X-rays.

Theoretical calculations: In this work, the adsorption energies of different two lithium salts and solvent molecules for the lithium metal anode and between NCM523 were calculated using the VASP calculation software package.^[1]^ The lithium (110) surface was first selected as the adsorption surface with lattice parameters of a = b = 10 Å and c = 20 Å. All simulated interactions between the ionic real and valence electrons were described using the electronic wavefunction (PAW) of the ultrasoft pseudopotential, and the exchange correlation was processed using the generalised gradient approximation (GGA) method of the Perdew-BurkeErnzerhof generalised (PBE) potential calculation. The cut-off energy of the plane wave is 450 eV. The energy and force convergence thresholds for the optimised ion step are set to 1 × 10^-5^ eV and 0.01 eV/Å, respectively, and the SIGMA is 0.1 eV using the Monkhorst-Pack method to sample the k-points in the Brillouin zone. The resulting k-point grid is 2 × 2 × 1. It is used for geometry optimisation and calculation of electronic properties.

The adsorption energy Eads can be used to evaluate the adsorption behaviour and the Eads value is calculated as follows:

E_ads_=E_total_-（E_surface_+E_absorbate_）

In the equation, Etotal, Esurface and Eabsorbate are the total energy of the system, substrate and adsorbed molecules, respectively.

Structural optimisation and LUMO and HOMO calculations of LiTFSI, LiNO_3_, PC, SN molecules at Hybrid B_3_LYP level were carried out using DMol_3_ module of Materials Studio.

Our MD simulations are carried out by using the large-scale atomic/molecular massively parallel simulator (LAMMPS).^[2]^ The molar ratios in the electrolyte were taken from those used in the experimental work. The all-atom force field used in the electrolyte is optimized potentials for liquid simulations (OPLSAA),^[3]^ where the parameters of Li^+^, NO_3_^-^ are taken from the literature.^[4]^  The Lennard-Jones potential was used to describe the non-bonding van der Waals forces between them, and the Lennard-Jones parameters between different atoms were calculated using the Geometric Mixing Rule with the following expression:

$\varepsilon_{ij}=\sqrt{\varepsilon_{i}\varepsilon_{j}}$ (1)

$\delta_{ij}=\sqrt{\delta_{i}\delta_{j}}$ (2)

and set the cut-off for the Lennard-Jones potential interaction to 10 Å.

The initial structure of the model was constructed by Packmol software.^[5]^ In the simulation process, the simulation step size is 1 fs, and the Velocity verlet algorithm^[6]^ is used to calculate the equations of motion, and the three directions of the simulated system are used with periodic boundaries In order to make the simulation model show the properties of the experimental electrolyte as much as possible, the conjugate gradient algorithm is used to minimise the energy of the system first, and in order to make the system reach the equilibrium state rapidly and reasonably, the In order to reach equilibrium quickly and reasonably, the system is annealed, and then both simulations are run under the NVT system for 4.0 ns to reach thermodynamic equilibrium, and finally, the density distribution of the substances in the electrolyte and the radial distribution function (RDF) are calculated by running the simulations under the NVT system for 1.0 ns.

**Figure S1** Long cycle test of LTS electrolyte under 3~4.3 V, 3~4.5V, 0.5 C conditions


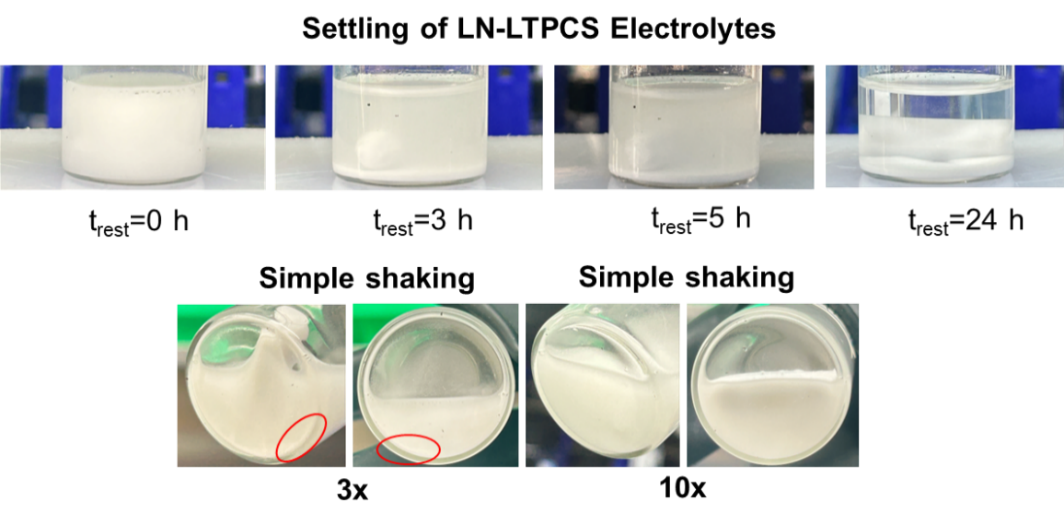


**Figure S2** The state of the LN-LTPCS suspension electrolyte resting at different times. Simple shaking by hand, the numbers represent 3x and 10x shaking of the LN-LTPCS suspension electrolyte after one day of settling.


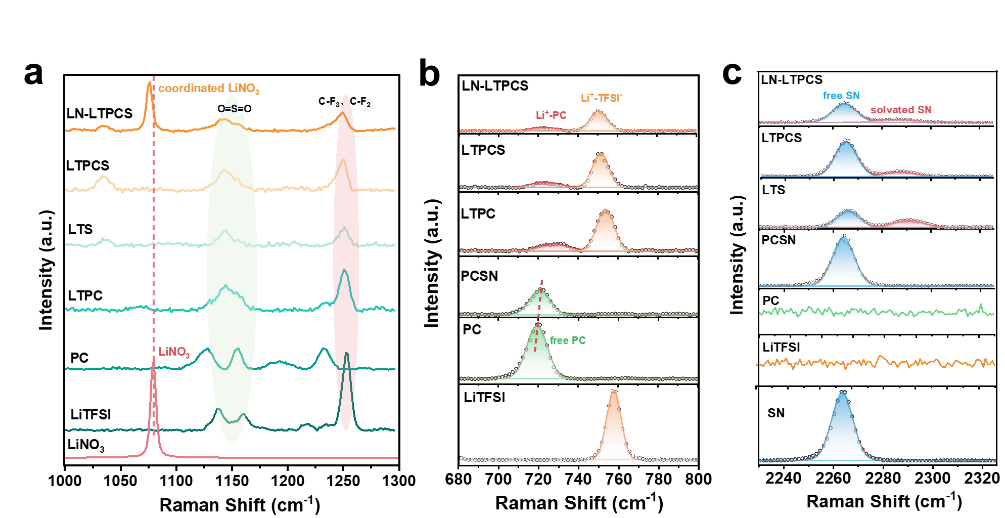


**Figure 3** Raman spectra of pure PC, SN, LiTFSI, LiNO_3_, PCSN, LTPC, LTS and LTPCS, LN-LTPCS electrolytes.


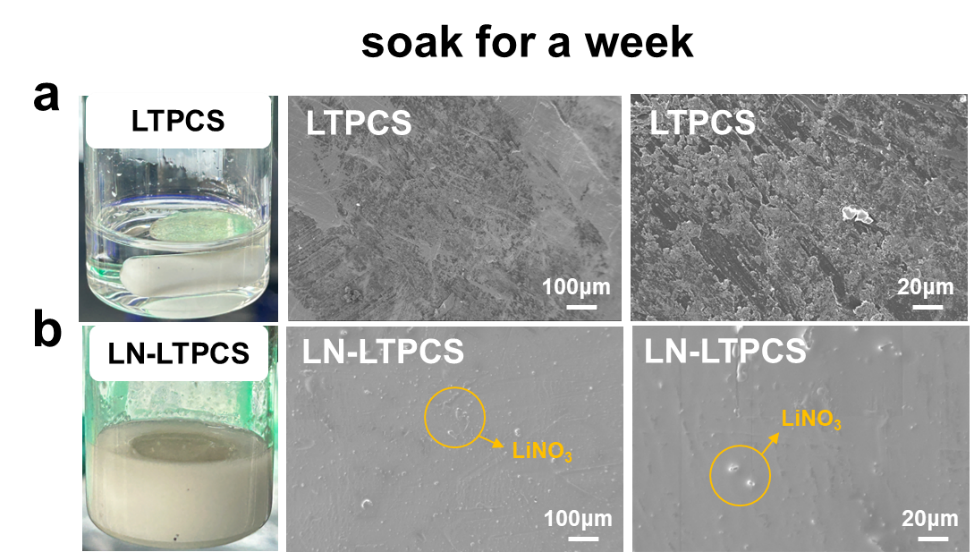


**Figure S4** SEM characterization of lithium metal after one week of immersion in LTPCS, LN-LTPCS respectively.

**Figure S5** Different states of electrolyte for Li||NCM523 at 4.3 V, 0.5 C charge/discharge test

**Figure S6** LN-LTPCS's evaluation of additives .

When the content of LiNO_3_ additive is lower than 4wt%, the cycling performance is relatively poor, indicating that the low content of LiNO_3_ can not form the adsorption deposition layer well, and the effect of inhibiting the side reaction at the electrode/electrolyte interface is poor. The effect of adding 10wt% is similar to that of adding 4wt%, considering the cost problem, so the additive content is finally fixed at 4wt% to obtain better performance.

**Figure S7** Li||LN-LTPCS||NCM523 cells repeat data of long cycle test at 25 ℃, 4.3 V, 0.5 C.


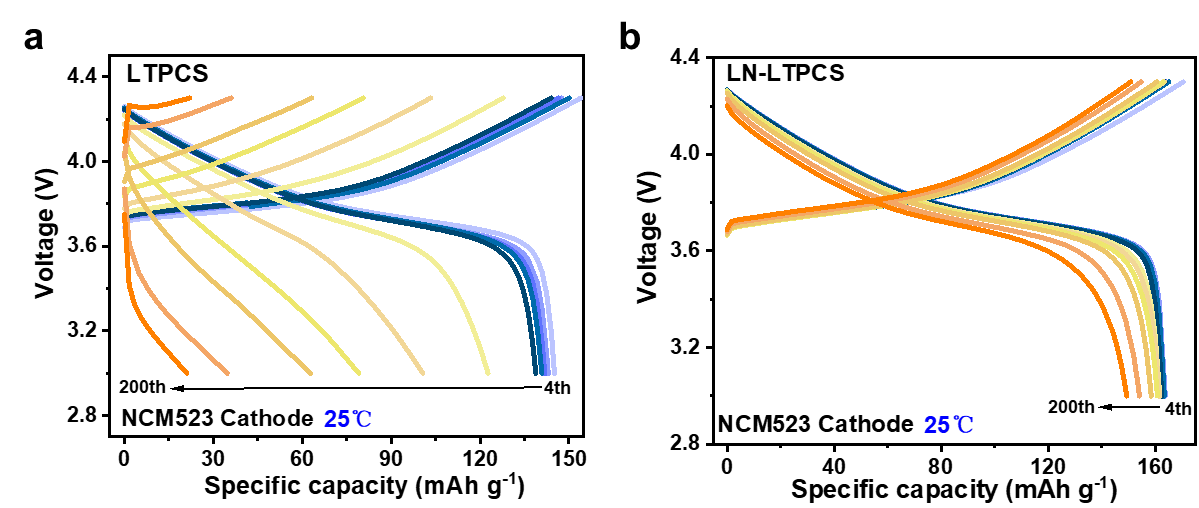


**Figure S8** Voltage specific capacity curve of Li||NCM523 at 25 ℃, 4.3 V, 0.5 C charging and discharging rate.

**Figure S9** Li||LN-LTPCS||NCM523 battery long cycle repeatable test at 25 ℃, 4.5 V, 1 C charge/discharge condition.


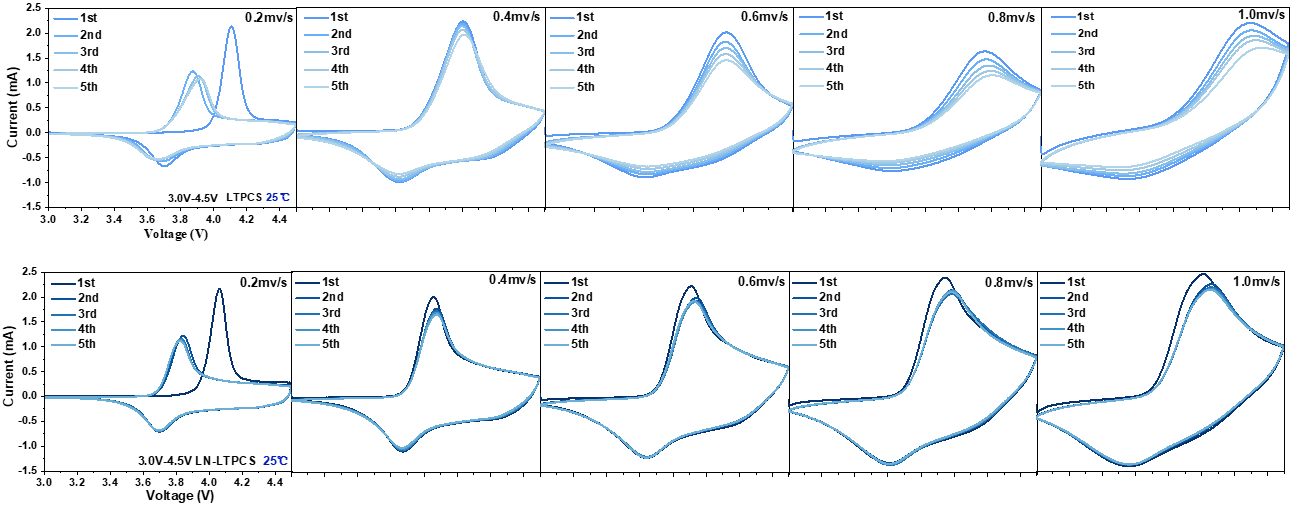


**Figure S10** CV test curves of Li||NCM532 batteries with different electrolytes were cycled five times at different scan rates of 0.2 mv/s, 0.4 mv/s, 0.6 mv/s, 0.8 mv/s and 1.0 mv/s in the range of 3~4.5 V high voltage.

**Figure S11** CV test curve of Li||LTPCS||NCM523 battery at different scan rates in the range of 3~4.5 V high voltage.


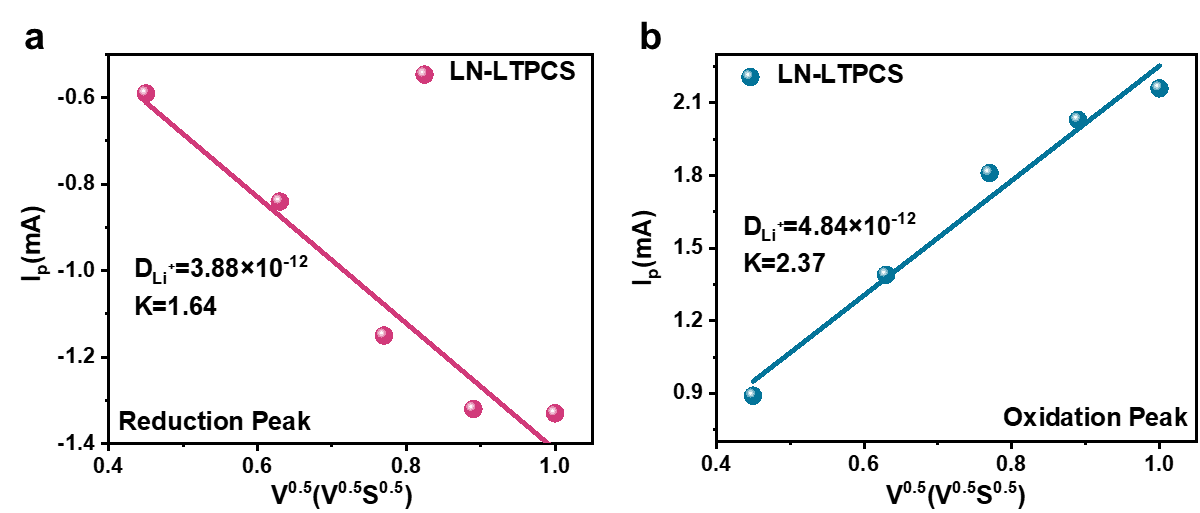


**Figure S12** Diffusion coefficient of LN-LTPCS electrolyte at high cut-off voltage (4.5 V).

**Figure S13** EIS after 150 cycles of different electrolyte systems at 4.5 V, 1 C

**Figure S14** XRD test of NCM523 after 150 cycles at high cut-off voltage (4.5 V, 1 C).

**Figure S15** Li||LN-LTPCS||NCM523 battery long cycle repeat test at 4.3 V, 60 °C.


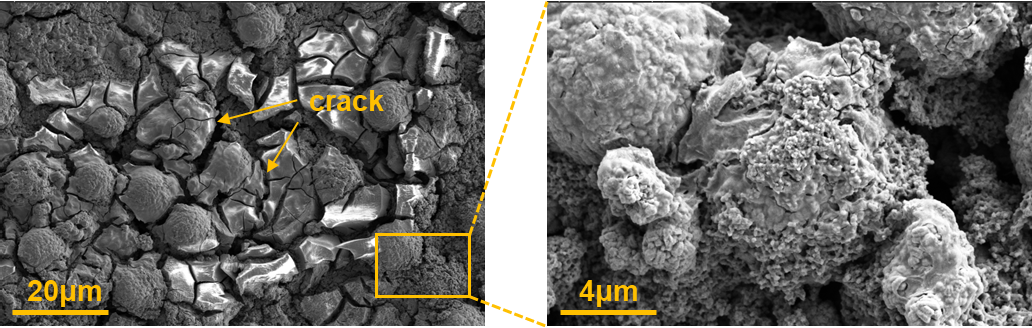


**Figure S16** SEM of NCM523 after 100 cycles of Li||LTPCS||NCM523 battery at 60 ℃.


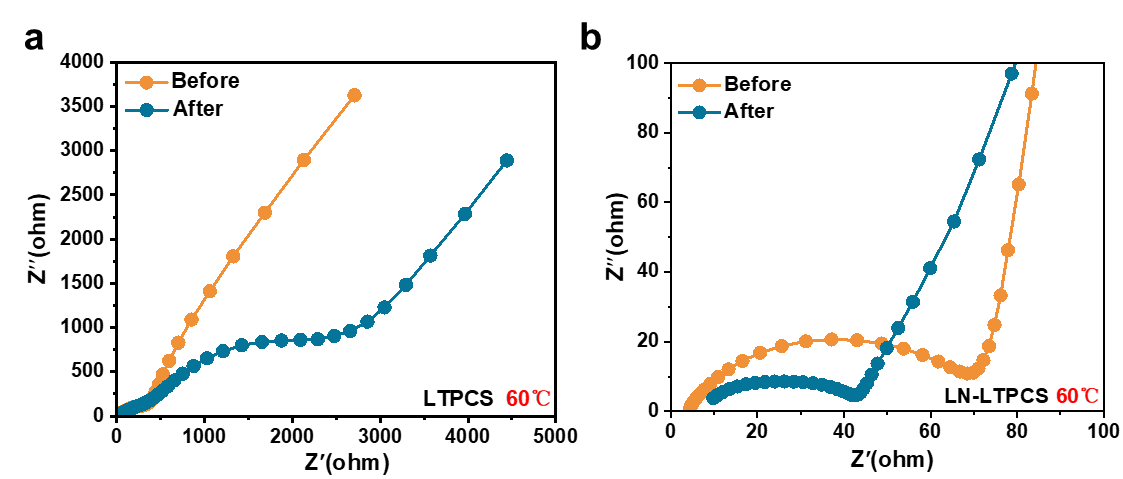


**Figure S17** Impedance test of Li||NCM523 battery before and after 100 cycles at high temperature (60 ℃)

**Figure S18** Li||LN-LTPCS||NCM523 battery low temperature (-10 ℃), 3~4.3 V, 0.2 C long cycle test

**Figure S19** Voltage specific capacity curve of Li||LN-LTPCS||NCM523 at -10 ℃, 4.3 V, 0.2 C charging and discharging rate.

**Supplementary Tables**

**Table S1**. Adsorption energy of each substance on the lithium metal (110）

| **Materialistic** | **E_total_ (eV)** | **E_surface_ (eV)** | **E_absorbate_ (eV)** | **E_ads_ (eV)** |
| --- | --- | --- | --- | --- |
| PC | -190.57 | -109.25 | -78.48 | -2.84 |
| SN | -176.50 | -109.25 | -65.77 | -1.48 |
| LiTFSI | -196.83 | -109.25 | -86.79 | -0.79 |
| LiNO_3_ | -143.69 | -109.25 | -27.17 | -7.27 |

**Table S2**. Adsorption energy of each substance on the NCM (003）

| **Materialistic** | **E_total_ (eV)** | **E_surface_ (eV)** | **E_absorbate_ (eV)** | **E_ads_ (eV)** |
| --- | --- | --- | --- | --- |
| PC | -957.83 | -879.28 | -78.48 | -0.07 |
| SN | -945.08 | -879.28 | -65.77 | -0.03 |
| LiTFSI | -966.31 | -879.28 | -86.79 | -0.24 |
| LiNO_3_ | -908.19 | -879.28 | -27.17 | -1.74 |

**References**

1. X. Shi, T. Zheng, J. Xiong, B. Zhu, Y.-J. Cheng and Y. Xia, *Acs Applied Materials & Interfaces*, **2021**, 13, 57107-57117.
2. P. Thompson, H. M. Aktulga, R. Berger, D. S. Bolintineanu, W. M. Brown, P. S. Crozier, P. J. i. t. Veld, A. Kohlmeyer, S. G. Moore, T. D. Nguyen, R. Shan, M. J. Stevens, J. Tranchida, C. Trott and S. J. Plimpton, *Computer Physics Communications*, **2022**, 271.
3. Jorgensen, William, L., Maxwell, David and S., *Journal of the American Chemical Society*, **1996**.
4. A. Shkatulov, B. Becit and D. Zahn, *Acs Omega*, **2022**, 7, 16371-16379.
5. L. Martinez, R. Andrade, E. G. Birgin and J. M. Martinez, *Journal of Computational Chemistry*, **2009**, 30, 2157-2164.
6. L. Verlet, *Health Physics*, **1967**, 22, 79-85.
